# Deep learning-based hyperspectral image correction and unmixing for brain tumor surgery

**DOI:** 10.1016/j.isci.2024.111273

**Published:** 2024-10-28

**Authors:** David Black, Jaidev Gill, Andrew Xie, Benoit Liquet, Antonio Di Ieva, Walter Stummer, Eric Suero Molina

**Affiliations:** 1 Department of Electrical and Computer Engineering, University of British Columbia, Vancouver, BC, Canada; 2 Engineering Physics, University of British Columbia, Vancouver, BC, Canada; 3 School of Mathematical and Physical Sciences, Macquarie University, Sydney, NSW, Australia; 4 Laboratoire de Mathématiques et de ses Applications, E2S-UPPA, Université de Pau & Pays de L’Adour, Pau, France; 5 Computational NeuroSurgery (CNS) Lab, Macquarie University, Sydney, NSW, Australia; 6 Macquarie Medical School, Macquarie University, Sydney, NSW, Australia; 7 Department of Neurosurgery, University Hospital Münster, Münster, Germany

**Keywords:** Bioinformatics, Cancer, Artificial intelligence

## Abstract

Hyperspectral imaging for fluorescence-guided brain tumor resection improves visualization of tissue differences, which can ameliorate patient outcomes. However, current methods do not effectively correct for heterogeneous optical and geometric tissue properties, leading to less accurate results. We propose two deep learning models for correction and unmixing that can capture these effects. While one is trained with protoporphyrin IX (PpIX) concentration labels, the other is semi-supervised. The models were evaluated on phantom and pig brain data with known PpIX concentration; the supervised and semi-supervised models achieved Pearson correlation coefficients (phantom, pig brain) between known and computed PpIX concentrations of (0.997, 0.990) and (0.98, 0.91), respectively. The classical approach achieved (0.93, 0.82). The semi-supervised approach also generalizes better to human data, achieving a 36% lower false-positive rate for PpIX detection and giving qualitatively more realistic results than existing methods. These results show promise for using deep learning to improve hyperspectral fluorescence-guided neurosurgery.

## Highlights

• Hyperspectral brain images are affected by tissue geometric and optical properties
• This can disrupt quantization of fluorophore content and invalidate measurements
• We introduce two semi-supervised deep learning models to correct for these effects
• The models outperform existing methods and can thus improve brain tumor surgery

## Graphical abstract

**Figure:**
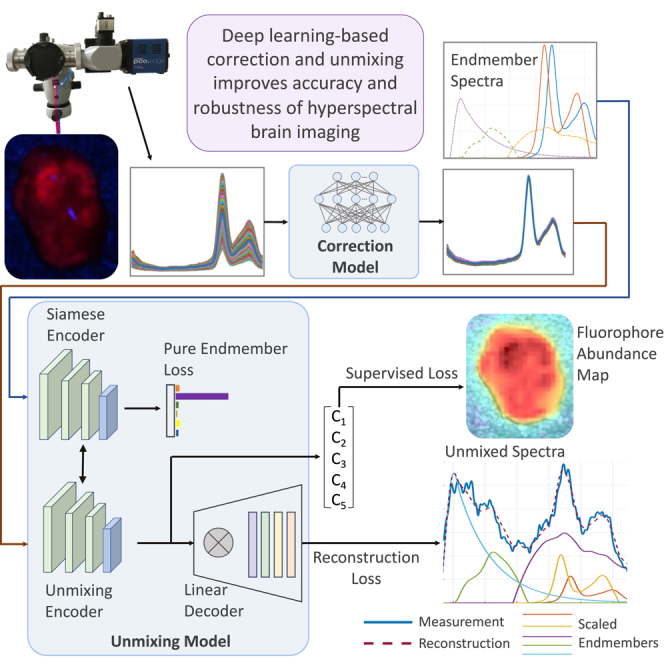

## Introduction

Due to their infiltrative growth, identifying glioma margins during brain surgery is extremely difficult, if not impossible. However, surgical adjuncts such as fluorescence guidance can maximize resection rates, thus improving patient outcomes.^1^^,^^2^ 5-Aminolevulinic acid (5-ALA) is a Food and Drug Administration-approved tissue marker for high-grade glioma.^3^ 5-ALA is administered orally 4 h before induction of anesthesia for fluorescence-guided resection of malignant gliomas; this drug is metabolized preferentially in tumor cells to protoporphyrin IX (PpIX), a precursor on the heme synthesis pathway.^4^ PpIX fluoresces bright red, with a primary peak at 634 nm, when excited with blue light at 405 nm. In this way, tumors that are otherwise difficult to distinguish from healthy tissue can sometimes be identified by their red glow under blue illumination. This allows for a more complete resection and thus improved progression and overall survival.^2^^,^^5^ However, the fluorescence is often not visible in lower-grade glioma or in infiltrating margins of tumors.^6^^,^^7^ In these cases, the PpIX fluoresces at a similar intensity to other endogenous fluorophores, known as autofluorescence, and remains indistinguishable.

Hyperspectral imaging (HSI) is, therefore, an active research area, as it allows the PpIX content to be isolated from autofluorescence. HSI systems capture three-dimensional data cubes in which each 2D slice is an image of the scene at a particular wavelength. A fluorescence intensity spectrum is obtained by tracing a pixel through the cube’s third dimension. Thus, the emission spectrum of light is measured at every pixel.^8^ This technology is used in many fields, including food safety and research,^9^ materials science,^10^ agriculture,^11^ and space exploration,^12^ as it provides rich spatial and spectral information without disturbing the system. In medical HSI, each spatial pixel contains a combination of fluorescing molecules or fluorophores. Assuming a linear model that neglects multiple scattering^13^ and other effects, the measured fluorescence spectrum at that pixel ($\Phi_{Fluo})$ is thus a linear combination of the emission spectra of K potentially present fluorophores ($\Phi_{Spec,k}$), also called endmember spectra,^14^ as shown in Equation 1 (ignoring noise). With *a priori* knowledge of the endmember spectra, linear regression techniques have been employed to determine the relative abundances ($c_{k}$) of the endmembers^15^ in a given spectrum.

(Equation 1) $$\Phi_{Fluo}=\sum_{k=1}^{K}c_{k}\Phi_{Spec,k}$$

**Figure 1:**
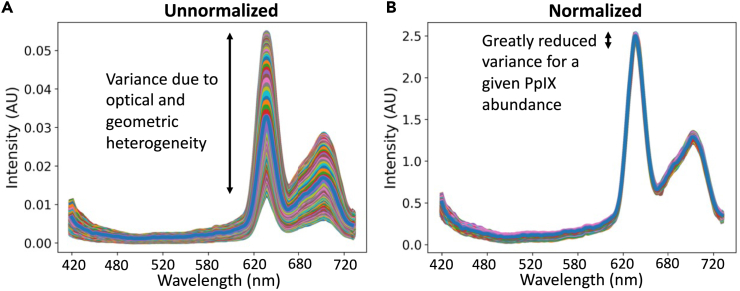
Typical attenuation correction of measured spectra from a phantom of constant PpIX concentration (A) shows the raw spectra with large variance, and (B) shows the normalized ones after correction. The variance in the magnitudes is greatly decreased.

Recent advances in HSI for fluorescence-guided surgery have increased our ability to detect tumor regions^8^^,^^16^ and even classify tissue types based on the endmember abundances.^17^^,^^18^ They have also been used to study 5-ALA dosage^7^ and timing of application,^6^ and to improve the imaging devices.^19^^,^^20^^,^^21^ However, these computations are extremely sensitive to autofluorescence, as well as to artifacts from the optical and topographic properties of the tissue and camera system. To mitigate the latter issue, the measured fluorescence spectra are corrected to account for heterogeneous absorption and scattering by using the measured spectra under white light illumination at the same location. With white light excitation, there is no fluorescence, so the measured spectra depend only on the heterogeneous tissue properties. The spectra can thus be used to correct for these variations. One common method for attenuation correction, called dual-band normalization, involves integrating over two portions of these spectra, raising one to an empirical exponent, and multiplying them to determine a scaling factor.^22^ While effective in phantoms,^23^ we have found this method to be of limited use in patient data.^16^ The pixels are also corrected for their distance from the objective lens since further pixels appear dimmer than closer ones.^24^^,^^25^ Other methods are also relatively simplistic, linear, and not based on human data.^26^ They are thus unable to account for nonlinear effects such as multiple scattering,^13^ the dual photostates of PpIX,^4^^,^^27^ and fluorescence variation due to pH and tumor microenvironment,^16^ nor can they entirely correct for the inhomogeneous optical properties of the tissue.^16^ These effects may also include wavelength-dependent absorption and scattering variations, which are unmodeled when using a single scaling factor. An example of attenuation correction is shown in Figure 1.

**Figure 2:**
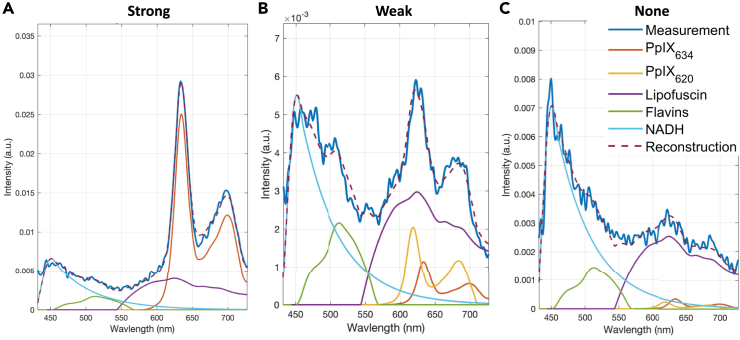
Example unmixing of three spectra with different PpIX content (A–C) Sample unmixing of three spectra with strong (A), weak (B), and very weak (C) PpIX content. The blue line is the measured spectrum, while the purple dashed line is the fit. The other spectra are the endmembers, scaled according to their abundance and summed to create the fitted spectrum. Unmixing enables recovery of PpIX abundance despite autofluorescence.

Once the spectra are corrected for optical and topological variations, they must be unmixed into the endmember abundances. In 5-ALA-mediated fluorescence-guided tumor surgery, these likely include the two photostates of PpIX,^4^^,^^27^ called PpIX_620_ and PpIX_634_, as well as autofluorescence from flavins, lipofuscin, NADH, melanin, collagen, and elastin,^15^^,^^28^ though there are usually only 3 or 4 endmembers present in any given spectrum.^29^ Previous work has commonly used non-negative least squares (NNLS) regression.^4^^,^^15^^,^^16^^,^^17^^,^^30^^,^^31^ This is simple and fast and guarantees non-negative abundances. Three example unmixings using NNLS are shown in Figure 2. Other papers have proposed Poisson regression^32^ to account for the theoretically Poisson-distributed photon emissions^33^ or various sparse methods to reduce overfitting and enforce the fact that there are usually only a few fluorophores present in each pixel.^14^ However, as mentioned before, all these methods assume linearity in the combination of the endmember spectra. Furthermore, they rely on the attenuation correction to be accurate.

Thus, performing the correction and unmixing in a single-step process that can handle the nonlinearity and complexity of the physical, optical, and biological systems described earlier would be beneficial. For this purpose, deep learning is particularly well suited because each HSI measurement produces a large volume of high-dimensional data. Indeed, deep learning has been explored in detail for HSI, as reviewed by Jia et al.,^34^ and for medical applications specifically.^35^^,^^36^ For brain tumor resection, the technique is very promising,^37^ and several studies have used support vector machines, random forest models, and simple convolutional neural networks (CNNs) to segment and classify tissues *in vivo*.^38^^,^^39^^,^^40^ Other approaches include majority voting-based fusions of k-nearest neighbors (KNNs), hierarchical k-means clustering, and dimensionality reduction techniques such as principal component analysis or t-distributed stochastic neighbor embedding.^41^^,^^42^ These papers used 61 images from 34 patients with a resulting median macro F1-score of 70% in detecting tumors. Rinesh et al. used KNNs and multilayer perceptrons (MLPs).^43^ The HELICoiD (Hyperspectral Imaging Cancer Detection) dataset,^44^ which consists of 36 data cubes from 22 patients, has been widely used. For instance, Manni et al. achieved 80% accuracy in classifying tumor, healthy tissue, and blood vessels using a CNN,^45^ and Hao et al. combined different deep learning architectures in a multi-step pipeline to reach 96% accuracy in glioblastoma identification.^46^ Other methods used pathological slides,^47^ with most mentioned based on small datasets.^48^

Given the small datasets, many of these papers have not yet had sufficiently good results to be clinically useful and likely do not generalize very well. This is partly due to the cost of labeling many hyperspectral images. As a result, modern architectures for medical image segmentation, such as U-Net,^49^ V-Net,^50^ or graph neural networks,^51^ have seen little use. Autoencoders^52^ or generative adversarial networks^53^ can use unsupervised learning for certain tasks to avoid the labeling problem but require large volumes of data. Jia et al. describe some approaches to overcome the lack of data in HSI,^54^ and self- or unsupervised approaches have been used in general HSI,^55^^,^^56^^,^^57^ but not in neurosurgery. In addition, these papers all represent end-to-end attempts to take a raw data cube containing high-grade glioma and output a segmentation. This approach is unlikely to generalize well to other devices, hospitals, or tumor types. Instead, a more fine-grained method may generalize better, in which the core steps of the process are individually optimized and rooted in the physics of the system. These steps include image acquisition, correction, unmixing, and interpretation of endmember abundances. The surrounding elements of device-specific processing can be kept separate. This separation also enables more flexible use of the results. For example, endmember abundances may be used to identify tumor tissue, classify the tumor type, or provide information about biomarkers such as isocitrate dehydrogenase (IDH) mutation, which is clinically highly relevant.^17^

As described before, classical methods for unmixing have some limitations. Therefore, research has explored deep-learning-based unmixing. Zhang et al. successfully applied CNNs to this task to obtain endmember abundances on four open-source agricultural HSI datasets.^58^ No similarly large dataset is available for brain surgery. Wang et al. used CNNs to obtain slightly better performance than non-negative matrix factorization on simulated and real geological HSI data.^59^ Others have used fully connected MLPs,^60^ CNNs,^61^ and auto-associative neural networks^62^ to unmix spectra without prior knowledge of the endmembers. However, these are not as effective when the endmember spectra are known, as in our case. An attractive solution called the endmember-guided unmixing network used autoencoders in a Siamese configuration to enforce certain relevant constraints, with good results.^63^ A review on-deep learning-based unmixing by Bhatt and Joshi shows that existing work is relatively minimal and preliminary.^64^ Much of the research does not use *a priori* known endmember spectra, and to the authors’ knowledge, none focuses on attenuation correction, neurosurgery, or HSI for fluorescence imaging.

This paper, therefore, describes a method of deep-learning-based correction and unmixing of HSI data cubes for fluorescence-guided resection of brain tumors. This improves on classical methods, can fit into any HSI pipeline in brain surgery, and gives generalizability and flexibility in the use of the endmember abundances. This is facilitated by the first use, to the authors’ knowledge, of modern architectures, including deep autoencoders and residual networks^65^ in HSI for brain tumor surgery. It is also the first use of a large and broadly diverse dataset for deep learning in HSI for neurosurgery, including 184 patients and 891 fluorescence HSI data cubes from 12 tumor types, all four World Health Organization grades, with IDH mutant and wild-type samples, and labeled solid tumor, infiltrating zone, and reactive brain (“healthy”) tissue. The models are optimized using phantoms and pig brain homogenate (PBH) data with known PpIX concentration. Due to the design’s physical underpinning, we show not only better quantitative results on these distributions but also improvements in generalizing to human data.

## Results

### Phantom and PBH results

**Figure 3:**
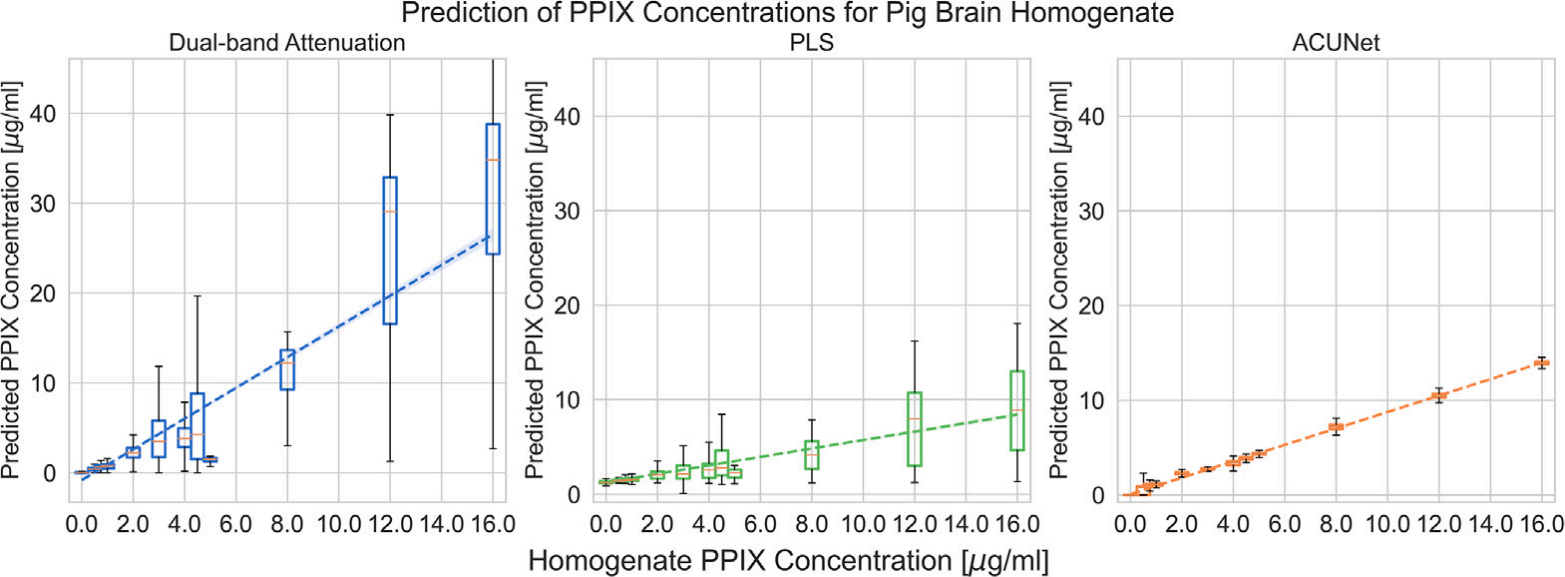
Linearity and variance of ACU-Net normalization and unmixing compared to previous methods Due to heterogeneous scattering and absorption, there is large variation in the measurements for a given known concentration. With ACU-Net, we see greatly improved linearity at a much lower variance and, consequently, a higher coefficient of determination than with the classical method and partial least-squares. These plots used the PBH data. The boxes are the interquartile range, with a line at the median, and the whiskers indicate the minimum and maximum.

Figure 3 shows the true and predicted PpIX concentration in PBH data using attenuation correction and hyperspectral unmixing network (ACU-Net) in contrast to the former approach, dual-band attenuation, and partial least-squares (PLS) regression.^66^ For PLS, all methods are evaluated using the same cross-validation data splits as described for ACU-Net training. The ACU-Net result has lower variance, indicating that the attenuation correction is effective. Additionally, the unmixed PpIX abundances are linear with the known abundances. Thus, the unmixing is also effective. In fact, the coefficient of determination for the PBH data was 0.97 using ACU-Net, compared to 0.82 with the benchmark method. The R value was similarly strongly improved for phantom data, as shown in Table 1.

**Table 1:** Comparison of proposed end-to-end learning-based normalization and unmixing compared to the benchmark dual-band normalization followed by non-negative least squares unmixing

	*Dual-band*	*ACU-Net*	*ACU-SA*	*PLS*	*MLP*
*Phantom data*	R = 0.93 RMSE = 3.77	R = **0.997** RMSE = **0.19**	R = 0.98 RMSE = 0.51	R = 0.93 RMSE = 0.35	R = 0.998 RMSE = 1.31
*Pig brain homogenate*	R = 0.82 RMSE = 4.17	R = **0.99** RMSE = **0.33**	R = 0.91 RMSE = 0.81	R = 0.67 RMSE = 2.10	R = 0.92 RMSE = 1.94

PLS and MLP approaches are also compared. The coefficient of determination between known and computed PpIX concentration is used for consistency with previous normalization work.^61^^,^^67^ For human data, no labels are available, so the reconstruction’s MSE (ReMSE) is used.

This shows that the supervised deep learning method can outperform classical methods. However, the semi-supervised ACU-SA method, too, shows a marked improvement in performance compared to the benchmark, with R values comparable to the supervised model. All the results, R values, and root-mean-square error (RMSE in μg/mL) for phantom and PBH data with the four methods are shown in Table 1.

In addition to PpIX quantification metrics, we have evaluated the runtime of each of the methods to validate whether the developed deep learning approach could be a potential step toward a real-time intraoperative technique. We observed, as shown in Table 2, that the mean runtime per pixel for the ACU-Net is greater than two times faster than the previous benchmark method.

**Table 2:** Comparison of runtime for each of the methods (mean ± standard deviation for a full 21,000-pixel test dataset, and per pixel)

*Runtime*	*Benchmark*	*ACU-Net*	*PLS*
*Total test set*	7720 ± 1,240 ms	3400 ± 303 ms	447 ± 45.3 ms
*Mean per pixel*	367.6 ± 59.0 μs	161.9 ± 14.4 μs	21.3 ± 2.2 μs

All differences are significant (*p* < 0.05).

### Extension to human data

Though the human data endmember abundances were not known, and no R or RMSE values could be computed, we nevertheless tested the methods on the human data to compare how well they generalize. For these tests, the models were trained on PBH data only, and the mean squared error (MSE) of the spectral reconstruction was measured on the PBH and human data. Good reconstruction does not guarantee accurate underlying endmember abundances, but it does provide some comparison of generalization. As shown in Table 3, ACU-Net and ACU-SA both generalize better to human data than the naive MLP does.

**Table 3:** Reconstruction MSE of the models trained on only PBH

	*ACU-Net*	*ACU-SA*	*MLP*
*PBH*	5.16e−5	5.48e−5	5.02e−5
*Human data*	5.95e−4	2.77e−4	7.87e−4

The dual-band method is excluded because it uses non-negative least squares so the MSE is minimal and there is no concept of generalization. ACU-SA and ACU-Net both generalize better to human data than a naive MLP.

Though the phantom and PBH results are promising, the critical question is whether the same results hold true in human data. While we currently do not have the true endmember abundances and thus cannot assess the performance quantitatively, we can observe that the average MSE reconstruction error of the ACU-Net is comparable to the benchmark method and much better than both the PLS and MLP. Note that the NNLS unmixing minimizes the sum of squared errors, so it is not possible to outperform it in this metric. It shows, however, that the model outputs are reasonable and close to optimal and that it generalizes better to human data than other existing methods.

In addition, the ability of the model to differentiate between healthy and tumor tissue is essential. Producing false-positive PpIX abundance readings, i.e., non-zero computed PpIX abundances where the actual abundance is zero, can be detrimental as they may cause erroneous resection of healthy brain tissue. Therefore, we measured the false-positive rate of the different methods on reactively altered human brain tissue, which should contain little to no PpIX. For these tests, the models were trained on the PBH data and tested on human data. The results in Table 4 show that the ACU-Net architecture outperforms existing methods.

**Table 4:** False-positive rate in human brain tissue—i.e., the percentage of spectra with zero expected PpIX abundance for which the method computed a non-zero value

*Method*	Dual-Band	MLP	ACU-Net
*False-positive rate*	13.1%	12.9%	8.39%

### Qualitative results

**Figure 4:**
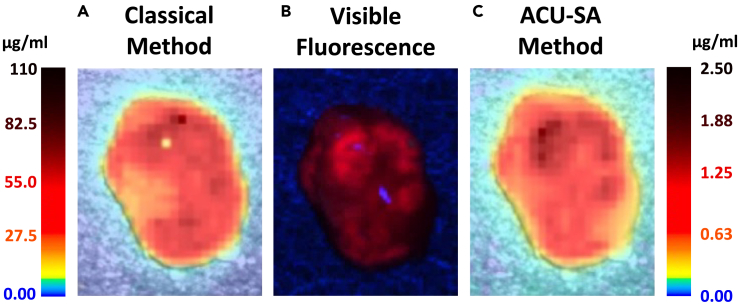
PpIX concentration map showing qualitative benefits of the proposed method PpIX concentration computed across a brain tumor sample using the classical method (A) and the ACU-SA (C). The deep-learning-based method shows a far more reasonable concentration range and better handles bright specular reflections in the top center of the sample. The visible fluorescence (RGB image, B) shows very similar patterns to the unmixing results.

Furthermore, differences in the output PpIX concentration maps are observed. In many cases where strong spots of specular reflection caused anomalous results in the dual-band normalization,^16^ the ACU-SA can remove the artifacts. This may be because the white light spectra in these cases were sometimes saturated, so the dual-band normalization would not sufficiently compensate, while a deep learning approach can better cope. In addition, previous papers have noted the difficulty of calibrating the unmixing output due to the nonlinear nature of PpIX fluorescence and the presence of more than one fluorescing state with different peak wavelengths.^4^^,^^16^^,^^27^ These factors lead, with the previous method, to unexpectedly large output PpIX concentrations in many cases. However, with the ACU-SA, the values appear far more reasonable, adhering more to expected values with less extreme variation. These factors are illustrated in Figure 4 and suggest that the deep learning approaches may have several benefits over classical methods for processing human data.

**Figure 5:**
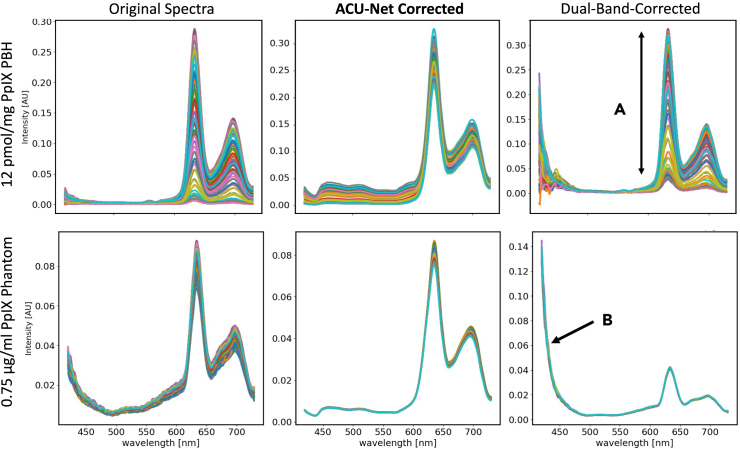
Corrected fluorescence emission spectra computed for both PpIX phantoms (top) and pig brain homogenate (bottom) using ACU-Net (middle) and the benchmark dual-band attenuation method (right) (A and B) The deep-learning-based correction shows lower variance given the same concentration (A) and better corrects for the blue light tail near the excitation wavelengths (B) than the classical dual-band method.

For ACU-Net, although we do not explicitly train to achieve a normalized fluorescence emission spectrum, we observe that the reconstructions do converge to a reasonable spectrum for samples of both phantom and PBH given the same PpIX concentrations. This is shown in Figure 5.

## Discussion

The results show that both supervised and semi-supervised learning outperform classical methods for correcting and unmixing hyperspectral brain tumor data. The performance of the semi-supervised method is promising for the field, as it shows that improved performance may be achieved without labeled datasets. Instead, data such as our human measurements can be used without ground truth abundance values. In this way, such models could be trained with large volumes of data and may generalize well to new human measurements. Additionally, it is shown that the ACU-Net method generalizes better to human data when trained on PBH data than existing classical or learning-based methods and that it achieves lower false-positive rates. Further work is required to continue improving the performance and to show quantitatively that it is effective on human data. This may involve chemical or histopathological assessment of samples co-registered to the HSI measurements, allowing for comparison of known absolute PpIX concentrations. The dataset should also be expanded to include non-tumor tissue to decrease false-positive endmember abundances. Additionally, enhancing the dataset with ground truth labels for the concentrations of both states of PpIX and the other fluorophores would better constrain the outputs of the relative abundance model outputs. Currently, only PpIX_634_ labels are available in phantom and PBH data.

For better generalizability and interpretability, it is best to separate the normalization and unmixing steps or at least have an intermediate state, which is the normalized spectra. Then, for example, the normalization network could be trained on phantom data with concentration labels and then attached to an unmixing network, which was trained unsupervised on human data. In this way, the whole model would generalize better since the unmixing cannot be trained on phantom data, which contain different endmember spectra than human brain, and the normalization is best trained with phantoms of constant, known concentration. This is achieved to a degree in this study but requires further investigation. Although ACU-Net and ACU-SA both achieved similarly high R value, we observed cases where the intermediate predicted normalized spectrum did not resemble a real measured spectrum. This indicates that the domain of the HU function the deep learning models learn is too large. Future work should find methods to constrain the shape of the predicted normalized spectrum more strongly to prevent the ACU-SA architecture from functioning as an end-to-end model and defeating the purpose of having a distinct normalization module. A related challenge is that the normalized spectrum is not known *a priori*. This is why both ACU-Net and ACU-SA rely on either an indirect or latent representation during training, which is not guaranteed to converge to true physical normalized spectrum. If phantoms are not sufficiently homogenous, the assumption that a common normalized spectrum exists is tenuous.

This study on the use of deep learning for analysis of hyperspectral images in fluorescence-guided neurosurgery invites several avenues of future research. These include integrating increasingly sophisticated models emerging from deep learning research, adding further constraints to enhance modeling accuracy, and enriching the available datasets to bolster the effectiveness of models. For example, to enable supervised learning on human data, mass spectrometry could be used to determine ground truth labels. It is also likely that spatial interactions between adjacent pixels in the hyperspectral images, which are currently not accounted for in our models, exist. Notably, relevant studies, including those using deep learning models for unmixing and otherwise analyzing hyperspectral images, have demonstrated improved performance when examining larger image regions instead of individual pixels. Therefore, adopting a model similar to ACU-SA but using a 2D CNN to account for spatial information can enhance correctional capabilities. This would likely improve the spatial smoothness of the abundance overlay plots and better handle localized artifacts such as bright reflections, as shown in Figure 4.

Another promising direction for future research is the integration of product and quotient relations into deep learning models. Previous studies^22^^,^^68^^,^^69^ have successfully utilized scaling factors that multiply or divide the measured fluorescence emission spectra for normalization. However, standard deep neural networks (DNNs) are better at capturing additive and non-linear relationships rather than direct multiplicative or divisive interactions. Incorporating multiplication or division operations directly or explicitly transforming them to log space into the model’s architecture could enable a DNN to represent these simpler analytical relations more efficiently, thus reducing the likelihood of overfitting and potentially offering a more accurate model of reality. However, it is essential to exercise caution regarding non-differentiability when incorporating these operations, as they can pose challenges in the gradient-based optimization process typically used in training DNNs.

### Limitations of the study

As outlined in the Discussion, there are several limitations of the current study that warrant future research. The primary limitation is that there were no ground truth labels for the human data, so performance in humans had to be assessed indirectly. Furthermore, the data are *ex vivo* and the imaging device is slow, so intraoperative use of the models *in vivo* will require more evaluation and potentially adjustment of the models for use with snapshot hyperspectral devices. The models themselves did not utilize spatial information, which would likely improve performance. Additionally, there were some cases where the intermediate predicted normalized spectrum did not resemble a real measured spectrum, so further improvement of the models would be beneficial.

### Conclusion

This paper has introduced two deep learning architectures that outperform prior methods for attenuation correction and unmixing of hyperspectral images in fluorescence-guided brain tumor surgery. The architectures explicitly enforce adherence to physical models of the system and condition on prior knowledge of the present endmember spectra, thus retaining some of the reliability and explainability of classical methods. Furthermore, the second introduced architecture can be trained in a semi-supervised manner, which allows the use of unlabeled human data and encourages better generalizability. The developed methods greatly improve the efficacy of the spectral correction and subsequent unmixing, decreasing unwanted variance and increasing the linearity of the estimated endmember abundances with respect to the expected abundances. They also decrease false-positive PpIX measurements and generalize better to human data than existing methods. These models will thus enable more accurate classification of brain tumors and tumor margins for intraoperative guidance in future work.

## Resource availability

### Lead contact

Further information and requests for resources and data should be directed to and will be fulfilled by the lead contact, Dr. Eric Suero Molina (Eric.Suero@ukmuenster.de).

### Materials availability

This study did not generate new unique reagents.

### Data and code availability

• The human data cannot be shared for privacy reasons, but phantom and PBH data may be shared upon reasonable request to the lead contact.
• The code for the described deep learning models is available on the repository linked in the key resources table.
• Further information about the human data is included in Table S1. Any additional information required to reanalyze the data reported in this paper may be made available from the lead contact upon request.

## Acknowledgments

We want to thank Carl Zeiss Meditec AG (Oberkochen, Germany) for providing us with the OPMI pico system and the BLUE 400 filter, as well as Sadahiro Kaneko, MD and Anna Walke, PhD for the assistance in the data collection. We also thank Projekt DEAL for enabling and organizing Open Access funding.

## Author contributions

Conception and design, D.B., A.X., J.G., and E.S.M.; acquisition of data, E.S.M.; statistical analysis and interpretation, D.B., A.X., J.G., B.L., and E.S.M.; drafting the article, D.B., A.X., and J.G.; critically revising the article, all authors; technical support, E.S.M.; study supervision, E.S.M.

## Declaration of interests

E.S.M. received research support from Carl Zeiss Meditec AG. W.S. has received speaker and consultant fees from SBI ALA Pharma, medac, Carl Zeiss Meditec AG, NXDC, and research support from Zeiss.

## STAR★Methods

### Key resources table

**Table:** 

REAGENT or RESOURCE	SOURCE	IDENTIFIER
**Biological samples**		

	Samples removed from patients undergoing brain tumor surgery with fluorescence guidance at the University Hospital Muenster	

**Software and algorithms**		

	https://github.com/dgblack/acunet_glioma	

### Experimental model and study participant details

Since the presented models require a mix of labeled and unlabeled data, three datasets were used in this paper: (1) brain tissue phantoms were created using known concentrations of PpIX, (2) PBH was spiked with known concentrations of PpIX, and (3) human brain tumor tissue was extracted during surgery and imaged *ex vivo*. All samples were measured on the same HSI device at the University Hospital of Münster, described below.

For phantoms, PpIX was mixed with Intralipid 20% (Fresenius Kabi GmbH, Bad Homburg, Germany) and red dye (McCormick, Baltimore, USA) in dimethyl sulfoxide (DMSO; Merck KGaA, Darmstadt, Germany) solvent to simulate the scattering and absorption, respectively, in human tissue, as described by Valdes et al..^22^^,^^23^ The PpIX concentrations were (0.0, 0.2, 0.6, 1.25, 2.5 μg/mL). By varying the other components, the following optical properties were achieved: absorption at 405 nm: μ_a, 405 nm_ = 18, 42, 60 cm^−1^; reduced scattering at 635 nm: μ’_s, 635 nm_ = 8.7, 11.6, 14.5 cm^−1^. More details are found in previous work.^16^

#### *Ex vivo* animal material

For the PBH, pig brain was obtained from a local butcher and separated into anatomical sections of cerebrum, cerebellum, hypothalamus, and brain stem/spinal cord. The tissue was washed with distilled water, cut into 10 × 10 × 10 mm pieces, and homogenized using a blender (VDI 12, VWR International, Hannover, Germany). The pH was controlled using 0.5 M tris(hydroxymethyl)aminomethane (Tris-base, Serva, Heidelberg, Germany) buffer and hydrochloric acid (HCl, Honeywell Riedel–de Haen, Seelze, Germany). For each sample, 200 to 600 mg of the homogenates were spiked with PpIX (Enzo Life Sciences GmbH, Lörrach, Germany) stock solution (300 pmol/μL in DMSO) to the desired concentrations (0.0, 0.5, 0.75, 1.0, 2.0, 3.0 and 4.0 pmol/mg) and homogenized using a vortex mixer. The PBH samples were placed in a Petri dish, making samples of about 4 × 4 × 2 mm. Approval for experiments with pig brains was given by the Health and Veterinary Office Münster (Reg.-No. 05 515 1052 21). More details about the PBH are available in previous work.^16^

#### Human participants

The human data used in this study was measured over six years (2018–2023) at the University Hospital Münster, Münster, Germany. Patients undergoing surgery for various brain tumors were given a standard dose of 20 mg/kg of 5-ALA (Gliolan, medac, Wedel, Germany) orally 4 h before induction of anesthesia. All procedures performed in these studies followed the ethical standards of the institutional and/or national research committee and with the 1964 Helsinki Declaration and its later amendments or comparable ethical standards. All experiments and clinical data analysis were approved by the local Ethics Committee (2015-632-f-S and 2020-644-f-S), and informed consent was obtained from all patients.

Tissue resected by the surgeons was immediately taken to the hyperspectral imaging (HSI) device and imaged *ex vivo* before being given to pathology. Each tissue sample measurement produced one data cube which on average contained approximately 623 spectra. In total, data cubes were measured for 891 biopsies from 184 patients, resulting in 555666 human brain tumor spectra. The tumor types are shown below.

**Table:** 

Category	# of Data Cubes	# of Patients
***TissueType***	*632*	*130*
Pilocytic Astrocytoma	5	2
Diffuse Astrocytoma	60	17
Anaplastic Astrocytoma	51	10
Glioblastoma	415	77
Grade II Oligodendroglioma	24	5
Ganglioglioma	4	2
Medulloblastoma	6	2
Anaplastic Ependymoma	8	2
Anaplastic Oligodendroglioma	4	1
Meningioma	37	8
Metastasis	6	2
Radiation Necrosis	20	4
***Margins (Gliomas)***	*288*	*67*
Reactively altered brain tissue	100	22
Infiltrating zone	57	18
Solid tumor	131	27
***WHO Grade (Gliomas****)*	571	*119*
Grade I	9	3
Grade II	84	20
Grade III	57	15
Grade IV	421	81
***IDH Classification***	*411*	*76*
Mutant	126	26
Wildtype	285	50

Of the 184 patients, 56.7% identified as male and 43.3% female. The ages ranged from 1 to 82, with a mean of 51.6 and median of 55. No meaningful difference in any of the endmember abundances was found as a function of age or sex.

### Method details

#### Architecture

Two neural network (NN) architectures were developed and tested in Python: a supervised model called ACU-Net and a semi-supervised autoencoder model called ACU-SA, inspired by EGU-Net.^63^ Given the data’s characteristics, we employ a 1D deep Convolutional Neural Network (1D CNN) architecture since neighboring wavelengths of the fluorescence and white-light spectra exhibit more correlation than those farther apart. This spatial and spectral correlation aligns well with the inductive bias inherent in CNNs. Additionally, we leverage residual connections, which allow for bypasses of certain layers^70^ and have been demonstrated to be a robust heuristic choice that improves the quality of learned features.^71^

For both models, the input data is $X\in R^{m\times n\times 2}$ where n is the number of spectra and m is the number of wavelength samples in each spectrum. We use the fluorescence emission spectrum $\Phi_{Fluo}\in R^{m}$, which is captured while exciting the region with light at λ=405 nm, and the white light reflectance spectrum $\Phi_{Ref}\in R^{m}$, which is captured while illuminating the region with broadband white light as explained in the Introduction. The two spectra are stacked to form a two-channel input spectrum, which utilizes the locality bias of the CNN. Let K be the number of known endmember spectra. The matrix whose columns are the endmember spectra is $B=\left[\begin{array}{ccc} \Phi_{spec,1} & \cdots & \Phi_{spec,k} \end{array}\right]\;{\in R}^{m\times K}$.

The HSI attenuation correction aims to correct the fluorescence emission spectra so that those originating from samples with equal fluorophore concentration have equal magnitudes irrespective of local optical or geometric properties. In other words, the goal becomes to minimize the variation between spectra of equal fluorophore content. Suppose there is an ideal corrected spectrum, $\Phi_{c}$, which is the pure emission of the fluorophores with all effects corrected for. Then, the correction seeks to minimize the variance between the predicted fluorescence spectra ${\hat{\Phi }}_{fluo,i}$, and $\Phi_{c},i\in \left[1,n\right]$. Thus, we use the mean squared error (MSE = $\frac{1}{n}\sum_{i=1}^{n}{\left\|{\hat{\Phi }}_{fluo,i}-\Phi_{c}\right\|}^{2}$) for the proposed models when predicting the true fluorescence emission spectra. Note $MSE\left(x\right)=Bias{\left(x\right)}^{2}+Var\left(x\right)$^72^, so minimizing this objective function does indeed minimize the variance between the predicted and the true normalized spectra. For models in which the abundances are output rather than reconstructed spectra, i.e., the output is the $c_{k}$ from Equation 1 rather than the $\Phi_{fluo}$, MSE is also used.

##### ACU-Net

The Attenuation Correction and Unmixing Network (ACU-Net) is a 1D CNN with four residual blocks, each containing 2–3 same-convolutions, each followed by a small max pooling layer to reduce the dimensions of the feature maps. Between each residual block, there is a convolution layer that approximately doubles the number of feature channels. A kernel size of 5 is used in the early layers and 3 in the later ones. The output of the convolutional layers is inputted to three fully connected layers. The architecture is shown in Figure 6. The white light and fluorescence emission spectra are stacked, so convolutions are performed together, as described above.

**Figure:**
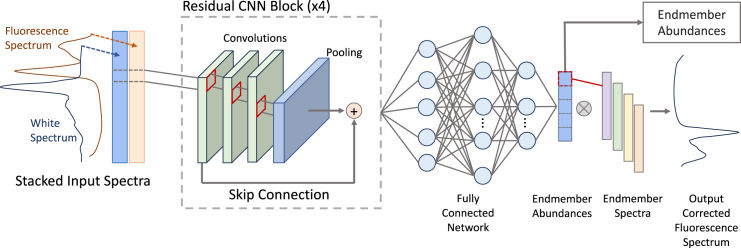
Spectrally informed attenuation correction and hyperspectral unmixing network architecture The inputs are the fluorescence and white-light spectra (orange and black, respectively, on the far left).

The goal of the ACU-Net is to learn the mapping $f:R^{m\times 2}\to R^{K}$ from the raw measured spectrum to the absolute endmember abundance vector, z, which includes PpIX_620_, PpIX_634_, and three primary autofluorescence sources: lipofuscin, NADH, and flavins.^15^ Other autofluorescence may be present,^28^ but these 5 spectra have been shown to fit well.^15^ This mapping is shown in Equation 2. The ground truth absolute PpIX concentration, denoted $c_{PPIX}$, is known for the phantoms and is known on average for the PBH, as described by Walke et al.^16^

(Equation 2) $$z=f\left(\Phi_{Fluo},\Phi_{Ref}\right),z\in R^{K}$$

Ground truth abundances are not, however, known for human data. Thus, a second loss - the reconstruction loss - is also considered with the aim of better generalization to human data. Let the relative abundance vector be $\hat{z}=\frac{z}{{\left\|z\right\|}_{2}}\text{.}$ We define the normalized reconstructed spectrum ${\hat{\Phi }}_{Fluo}=\sum_{k=1}^{K}{\hat{z}}_{k}\Phi_{spec,k}=B\hat{z}$, which should be as close as possible to the true corrected spectrum, $\Phi_{c}$ described above. The normalization is important to avoid bias toward strong PpIX spectra which have much larger magnitude than weak ones. The mapping f thus also aims to minimize $MSE\left({\hat{\Phi }}_{fluo,i}-\Phi_{c}\right)$. There is no known ground truth spectrum $\Phi_{c}$. Instead, ACU-Net uses the non-corrected $\Phi_{Fluo}$, hypothesizing that by training on a large and diverse dataset, ${\hat{\Phi }}_{fluo}$ will converge toward an average representation that best characterizes $\Phi_{c}$. Additionally, it is essential to note that the learned ${\hat{\Phi }}_{fluo,i}$ will not fit as precisely as methods employing least squares (LS), which are mathematically optimal and tend to overfit. Instead, by utilizing a deep neural network (DNN), we aim to more effectively learn the corrected fluorescence spectrum $\Phi_{C}$, and the abundances underlying the noisy measurement.

Using a rectified linear unit (ReLU) activation function at the output of the final layer enforces the non-negativity constraint on the relative abundance values. Finally, we use a weighted loss to train the model to minimize both the error in predicted concentration and the reconstruction error. Since the two objectives are of different scales and it is unknown how the structure of our architecture may affect the learning, the loss weights are also parameterized by considering the homoscedastic uncertainty of each task as outlined by Kendal et al.^73^ Denoting $\sigma_{C}$ and $\sigma_{rec}$ as the learned parameters for weighing the concentration prediction and spectrum reconstruction components of the architecture, we write the total loss function for one measured spectrum in Equation 3.

(Equation 3) $$L=\frac{1}{2\sigma_{C}^{2}}{\left(z_{1}-c_{PPIX}\right)}^{2}+\frac{1}{2\sigma_{rec}^{2}}{\left\|Bz-\Phi_{fluo}\right\|}_{2}^{2}+\log\left(\;\sigma_{C}\sigma_{rec}\right)z=f\left(\Phi_{Fluo},\Phi_{Ref}\right)$$

##### ACU-SA

The challenge with ACU-Net is that it requires ground-truth abundance labels, which are only available for phantom data. Therefore, we also propose a semi-supervised model. Attenuation Correction and Unmixing by a Spectrally-informed Autoencoder (ACU-SA) is similar to the EGU-Net,^63^ using an endmember-guided semi-supervised approach to the unmixing process. ACU-SA consists of two main components: one for hyperspectral unmixing (HU) and one explicitly for normalization. The HU portion consists of a Siamese autoencoder architecture, as shown in Figure 7, outlined in green. The objective of this portion is to learn a mapping $:R^{m}\to R^{K}$ , from the normalized fluorescence spectrum to the absolute endmember abundances, like ACU-Net. However, unlike ACU-Net, this portion takes the attenuation-corrected fluorescence emission spectrum as an input rather than the stacked raw spectra and, through its autoencoder structure, unmixes and reconstructs it. The HU component has the same architecture as the ACU-Net. Then, ACU-SA also includes a standalone CNN normalization model (blue outline in Figure 7) whose objective is to learn the mapping $g:R^{m\times 2}\to R^{m\times 1}$, from the two captured spectra to an intermediate representation, which we train to be the normalized/corrected fluorescence spectrum. Together, the normalization model takes the stacked white and fluorescence spectra, performs the attenuation correction, and feeds into the HU autoencoder network, which unmixes it into the absolute endmember abundances. Our normalization model is a shallow 1D-CNN with four convolutional layers and no residual blocks.

**Figure:**
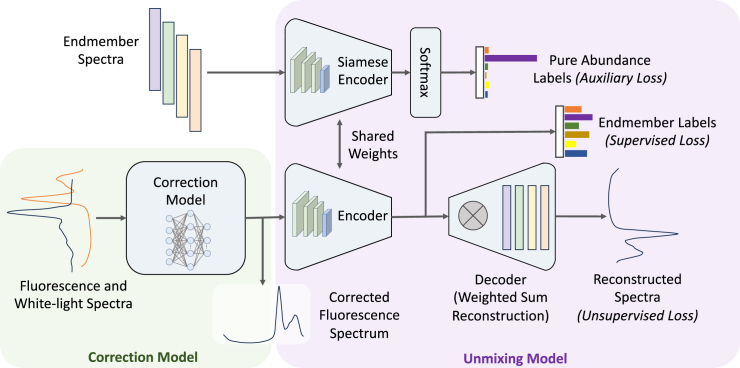
Endmember-guided normalization (blue outline) and unmixing (green outline) network for semi-supervised learning through an autoencoder architecture The endmember embeddings can be used for a supervised loss, while the autoencoder reconstruction is used for semi-supervised training. A second encoder with identical parameters is used with the pure endmember spectra as input, to condition the network on the known endmember spectra.

For supervised learning, the output embeddings from the encoder can be compared to known abundances using the MSE. Otherwise, the decoder reconstructs the spectrum from the abundance values so it can be compared to the input spectrum to obtain an unsupervised reconstruction loss. As with ACU-Net, the decoder uses the output embeddings as weights in the linear combination from Equation 1. Thus, the decoder has fixed parameter weights to ensure the encoders embeddings represent the real endmember abundances.

A twin encoder with shared weights to the HU encoder is used with a SoftMax output and evaluated with a cross-entropy loss. The pure endmember spectra are input to this network, and the output should ideally be a one-hot vector. For example, if the second endmember is input, the unmixing should output zero for all the endmembers except the second, which should be one. In this way, the independence of the endmember spectra is enforced, and we ensure that the output embeddings each correspond to only one endmember. This conditions the network on our *a priori* knowledge of the endmember spectra and has been shown to be effective in deep neural networks for HU.^63^

ACU-SA is trained in two stages. First, the HU network is trained to learn f for the PBH and homogenate datasets. We use a small NN as opposed to other linear and nonlinear HU methods such as least squares and non-negative matrix factorization because it is fully differentiable and easily be incorporated with the other components in ACU-SA. There is also evidence that DNN autoencoders are more robust to environmental noise for HU.^63^^,^^74^ Since this stage is fully self-supervised, we can augment the training data with synthetic data composed by creating random linear combinations of the known endmember spectra plus noise to help the HU module learn unmixing more effectively, and we can use unlabeled human data. The loss function used for training the HU is given in Equation 4, where ${\hat{e}}_{k}\in R^{K}$ is all zeros with a 1 in the *k*^*th*^ element.

(Equation 4) $$L_{HU}=\frac{1}{2{K\sigma }_{EG}^{2}}\sum_{k=1}^{K}CE\left(f\left(\Phi_{spec,i}\right)\right)+\frac{1}{2\sigma_{rec}^{2}}{\left\|\text{Bz}-{\hat{\Phi }}_{fluo}\right\|}^{2}+\log\left(\;\sigma_{EG}\sigma_{rec}\right)\;z=f\left({\hat{\Phi }}_{fluo}\right)CE\left(\phi \right)=\log\frac{e^{\phi^{T}{\hat{e}}_{k}}}{\sum_{j\in \left\{1,\ldots ,K\right\}}e^{\phi^{T}{\hat{e}}_{j}}}$$

Here $\sigma_{EG}$ and $\sigma_{rec}$ are again learned loss weightings as used in ACU-Net. For the second stage of training, the weights of the HU module are frozen, and the normalization module is attached. Then, given a much smaller amount of data labeled with their PpIX concentrations, the full network can be trained, optimizing only the weights for the normalization module. The loss function for this stage is shown in Equation 5, where ${\left[x\right]}_{i}$ represents the *i*^*th*^ element of vector x.

(Equation 5) $$L={\left({\left[f\left(g\left(\Phi_{fluo,}\Phi_{ref}\right)\right)\right]}_{1}-c_{PpIX}\right)}^{2}$$

The models are physics-informed because they take advantage of the spatial and spectral correlation in the measurements and are optimized with respect to abundances of known fluorescence emission spectra of the predominant fluorophores in brain tissue. Additionally, compared to other works using DNN models which directly perform semantic segmentation of tissue, our model outputs a prediction for a definite and physical quantity. Furthermore, our approach splits correction and unmixing into two modules which can be trained or modified individually, and conditions the unmixing autoencoder to correspond directly with the known endmembers by utilizing a Siamese network and decoding through an explicit weighted sum of the endmember spectra.

#### Dataset

Samples of each type (human, PBH, phantom) are shown in Figure 8. All the samples from these sources were imaged using an HSI device previously described, and some were used in prior research into 5-ALA dosage and timing, tissue type classification, and optimization-based unmixing.^6^^,^^7^^,^^8^^,^^15^^,^^16^^,^^17^ The sample was illuminated with white light to capture the white light spectra, blue light from a 405 nm LED for the fluorescence spectra, and not at all for dark spectra, which were used to remove the dark noise of the camera sensor. The reflected and emitted light was captured with a ZEISS Opmi Pico microscope (Carl Zeiss Meditec AG, Oberkochen, Germany) and passed through several low and high-pass filters to remove, for example, the brightly reflected blue excitation light. The light then passed through a liquid crystal tunable filter (Meadowlark Optics, Longmont, CO, USA) to a scientific metal oxide semiconductor (sCMOS) camera (PCO.Edge, Excelitas Technologies, Waltham, MA, USA). Data cubes were captured by sweeping the filter through the visible range from 421 to 730 nm in 3 nm steps and capturing a 2048 x 2048-pixel grayscale image at every sampling wavelength. Each image had a 500 ms exposure time to ensure good signal-to-noise ratio even from faint fluorescence. Additionally, 10 x 10 regions of pixels were averaged to reduce noise. The microscope focus was such that each region was 210 × 210 μm in size.

**Figure:**
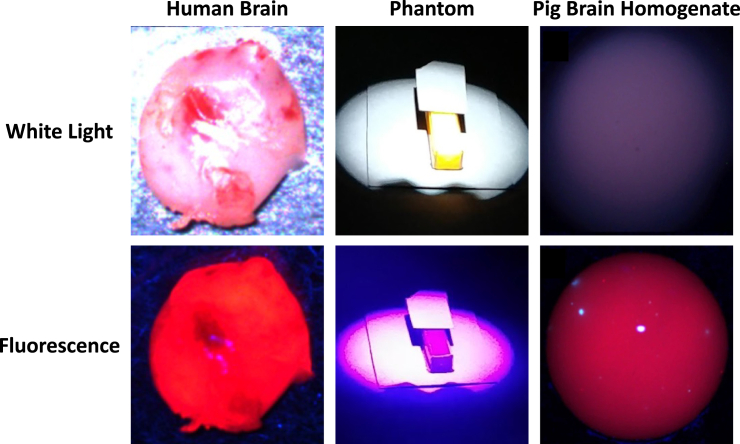
RGB images of typical samples of human brain, phantom, and pig brain homogenate, each under white light and blue light (fluorescence) illumination The PBH images are used under CC BY 4.0 license from ref. ^17^

Once captured, each data cube contained the sample of interest surrounded by background of the slide. Extracting the spectra from only the sample by manual segmentation is tedious, so classical computer vision techniques of edge and blob detection and morphological opening were used to detect the sample automatically. This was later augmented using a Detectron 2 model trained on our images.^67^ Within these selected areas, regions of 10 x 10 pixels were averaged to increase the signal-to-noise ratio, and as many non-overlapping regions as possible were extracted from the biopsy to ensure independent data samples. The spectra were then corrected for the filter transmission curves and wavelength-dependent sensitivity of the camera. Approximately 500–1000 spectra were measured from each biopsy.

In total, data cubes were measured for 891 biopsies from 184 patients, resulting in 555666 human brain tumor spectra. The human data is shown in Figure 9. The phantom data consisted of 9277 spectra, and the PBH samples were large and constituted 198816 spectra.

**Figure:**
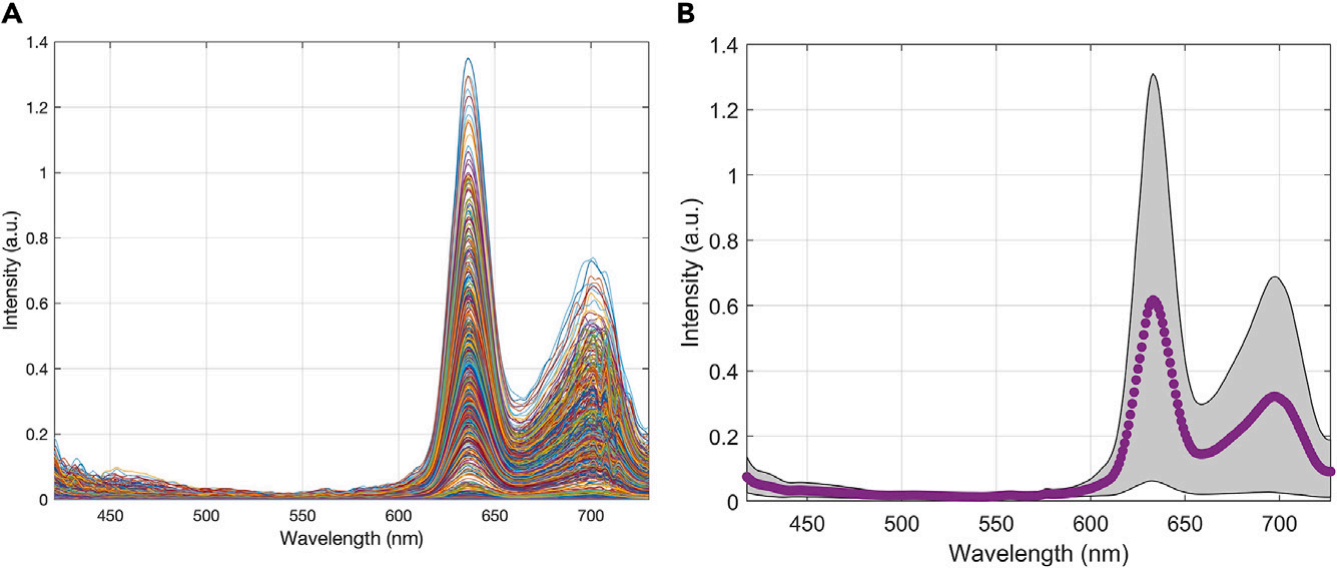
1,000 typical human fluorescence spectra were randomly sampled from the dataset of 555,666 total spectra These show clear PpIX content and vary widely in magnitude. (A) shows the spectra themselves while (B) shows the mean spectrum’s sample points with the range of one standard deviation in gray.

#### Experiments

Various tests were performed to determine the performance of the models on the dataset. As a baseline method or benchmark, the classical attenuation correction and unmixing procedures described in the Introduction were used, including dual-band normalization and nonnegative least squares unmixing. This is currently the most commonly used method in the field. Partial least-squares (PLS) regression^66^ and a multi-layer perceptron (MLP) model were also used for comparison. PLS is representative of other PCA-based methods commonly employed in many recent studies.^18^^,^^75^ The naive deep learning approach was an MLP with an input layer size of 610; the input fluorescence and white light spectra were horizontally stacked. The hidden layer sizes were 8, 5, and 8, with an output size of 310. The model was trained the same way as ACU-Net: the output at the size-5 hidden layer was optimized to the concentration labels, while the 310-dimensional final output was used to compute reconstruction loss. All other optimizer parameters, such as the batch size, learning rate scheduler, etc., were the same as for the ACU-Net.

To evaluate the performance, we used not only the MSE of the reconstructed spectra or calculated abundance vectors, but primarily the correlation coefficient (R) between the measured and ground truth PpIX concentrations. This should ideally be linear, so an R as close to 1 as possible is desired. In this way, the method can be calibrated with a single scaling factor. We also evaluated the runtime of each method since speed is important for real-time intraoperative imaging. These tests were carried out on a laptop with an Nvidia GeForce GTX 1050 GPU and an Intel Core i7-8550U CPU by running a Python timeit library test for a test set of 20 hyperspectral pixels and averaging over 12 runs.

To test the fully supervised ACU-Net, it was necessary to use the phantoms and PBH data, which had ground truth labels. It was possible to train the ACU-SA on human data. However, assessing its performance was difficult without known abundances, and thus comparing methods was impossible. Therefore, the ACU-SA was also trained on the phantom and PBH data for quantitative evaluation before using the human data for a qualitative analysis. For training the ACU-SA, each dataset was split approximately 85% and 15% into training and testing sets, respectively. The split was performed by sample of pig brain or vial of phantom rather than by pixel to avoid bias. All results presented below are on test data unseen during training. The models were trained using the AdamW optimizer with an adaptive learning rate that decreases on training loss plateau. No hyperparameter tuning was done.

### Quantification and statistical analysis

For statistical significance tests of the difference between two distributions, we used the two-sample Kolmogorov-Smirnov test (kstest2.m in MATLAB) because the data is continuous, and the test does not make assumptions about its distribution. A *p* value of less than 0.05 was considered statistically significant. Where a value is given in the paper as x±y, x is the mean and y is the standard deviation. For all implementation and testing of the deep learning models, Python was used with various packages including NumPy, SciPy, Scikit-image, PyTorch, and TimeIt.
