# Supplementary material for: Deep learning-based hyperspectral image correction and unmixing for brain tumor surgery
Source: iScience. 2024 Oct 28;27(12):111273. doi: 10.1016/j.isci.2024.111273 (PMC11613202; doi:10.1016/j.isci.2024.111273)
Supplement: Document S1. Table S1 [file mmc1.pdf]

iScience, Volume *27*

Supplemental information

Deep learning-based hyperspectral image correction and unmixing for brain tumor surgery

David Black, Jaidev Gill, Andrew Xie, Benoit Liquet, Antonio Di Ieva, Walter Stummer, and Eric Suero Molina

Supplemental Table S1: Human Brain Tissue

Category	# of Data Cubes	# of Patients
Tissue Type	*632*	*130*
Pilocytic Astrocytoma	5	2
Diffuse Astrocytoma	60	17
Anaplastic Astrocytoma	51	10
Glioblastoma	415	77
Grade II Oligodendroglioma	24	5
Ganglioglioma	4	2
Medulloblastoma	6	2
Anaplastic Ependymoma	8	2
Anaplastic Oligodendroglioma	4	1
Meningioma	37	8
Metastasis	6	2
Radiation Necrosis	20	4
Margins (Gliomas)	*288*	*67*
Reactively altered brain tissue	100	22
Infiltrating zone	57	18
Solid tumor	131	27
WHO Grade (Gliomas)	571	*119*
Grade I	9	3
Grade II	84	20
Grade III	57	15
Grade IV	421	81
IDH Classification	*411*	*76*
Mutant	126	26
Wildtype	285	50
